# Causal role of 731 immune cell types in atrial fibrillation: A bidirectional two-sample Mendelian randomization study

**DOI:** 10.1097/MD.0000000000046767

**Published:** 2025-12-19

**Authors:** Luofei Huang, Han Li, Quanzhi Lin, Jian Shi

**Affiliations:** a Liuzhou Municipal Liutie Central Hospital, Liuzhou, China; b Department of Internal Medicine, Liuzhou People’s Hospital, Liuzhou, China; c Department of Internal Medicine, The First Affiliated Hospital of Guangxi University of Science and Technology, Liuzhou, China; d Department of Internal Medicine, The People’s Hospital of Laibin, Laibin, China.

**Keywords:** atrial fibrillation, causal inference, immune cells, Mendelian randomization, sensitivity analysis

## Abstract

Mounting genetic evidence indicates that immune dysregulation may be a key driver in the development of atrial fibrillation (AF), yet the specific immune pathways involved remain poorly characterized. In this study, we applied advanced Mendelian randomization approaches to systematically assess the causal relationships between 731 precisely defined immune cell traits and susceptibility to AF. By integrating large-scale genetic datasets and employing rigorous statistical frameworks—including inverse variance weighting and weighted median analyses—we identified 2 immune signatures with significant causal associations. Genetically predicted increases in IgD^‐^CD38^dim B-cell frequency were associated with a higher risk of AF (odds ratio = 1.049, 95% confidence interval 1.024–1.075, *P* = .0001), whereas elevated CD25 expression on IgD^+^CD38^dim B cells conferred a protective effect (odds ratio = 0.974, 95% confidence interval 0.962–0.986, *P* = 1.87 × 10^‐5^). Both associations remained significant after stringent false discovery rate correction (post false discovery rate < 0.05). These findings provide the first genetic evidence linking specific B-cell regulatory mechanisms to AF pathogenesis, offering novel biological insights into the immune basis of atrial arrhythmogenesis. Beyond advancing mechanistic understanding, this work highlights the power of genetic epidemiology to uncover previously unrecognized immunomodulatory targets for AF prevention and therapy.

## 1. Introduction

Atrial fibrillation (AF) is the most prevalent cardiac arrhythmia, characterized by disorganized electrical activity in the atria, leading to irregular heart rhythms and an increased risk of stroke, heart failure, and other cardiovascular complications.^[1–3]^ The pathophysiology of AF is complex, involving both electrical and structural remodeling of the atrial myocardium. Approximately 10.55 million adults in the United States suffer from AF, a condition that significantly increases the risk of stroke, heart failure, myocardial infarction, dementia, chronic kidney disease, and mortality. Typical symptoms of AF include palpitations, shortness of breath, chest pain, presyncope, exercise intolerance, and fatigue. However, 10% to 40% of AF patients have no symptoms. Even so, the all-cause mortality rate of AF patients is still 1.5 to 2 times higher than that of the general population, mainly driven by AF-related complications instance, the development of stroke can further increase their mortality rate by 2 to 4 times.^[4]^ Recent studies suggest immune mechanisms as an additional contributor to this remodeling process. Key factors contributing to the initiation and maintenance of AF include atrial dilation, fibrosis, and conduction abnormalities.^[5,6]^ In recent years, growing evidence has highlighted the critical role of immune cells in the pathogenesis of AF.^[7,8]^

Immune cells play a pivotal role in the development and progression of various human diseases.^[9–13]^ The immune response in AF is thought to be triggered by various factors such as atrial stretch, ischemia, and oxidative stress.^[14]^ These stimuli activate immune cells, including macrophages, T cells, and dendritic cells, which infiltrate the atrial tissue.^[15]^ Immune cell infiltration releases pro-inflammatory cytokines and chemokines, driving inflammation and contributing to structural remodeling of the atrial myocardium.^[16,17]^ Macrophages contribute to tissue remodeling, while T cells promote inflammation and fibrosis via cytokines. Additionally, dendritic cells are implicated in the activation of both innate and adaptive immune responses, further exacerbating the progression of AF.^[5]^ This immune cell-mediated inflammation not only affects the electrical properties of the atrium but also facilitates fibrosis, which disrupts normal conduction pathways, creating a substrate conducive to AF. Therefore, understanding the specific role of immune cells in AF is essential for the development of novel therapeutic strategies targeting the immune system. Recent studies suggest that immunomodulatory therapies may reduce inflammation and prevent AF, underscoring the relevance of immune-related mechanisms in AF treatment.

Mendelian randomization (MR) represents a robust method for elucidating causal relationships in observational studies.^[18–21]^ By leveraging large-scale genomic data and genetic variants associated with immune cell types, MR offers profound insights into the potential therapeutic implications for modulating immune responses in AF patients.^[22,23]^ This study aims to leverage the strengths of MR to elucidate the causal relationships between immune cell subtypes and the risk of AF. It provides a novel perspective on AF pathogenesis and highlights potential immune-related targets for therapeutic intervention. Notably, this work carries substantial significance for both academic research and clinical practice. From an academic standpoint, it represents the first systematic investigation of the causal associations between 731 precisely characterized immune cell traits and AF susceptibility using advanced MR methodologies.

## 2. Materials and methods

### 2.1. Research and design scheme

In our research, MR analysis assessed the causal relationship between 731 immune cell types and AF. For MR causal inference to be valid, it must adhere to 3 fundamental assumptions: firstly, a strong correlation exists between genetic variation and the exposure of interest^[24]^; secondly, the association between genetic variation and exposure is independent of confounding factors^[25]^; thirdly, genetic variation impacts the outcome solely through the exposure pathway.^[26]^ Bidirectional MR was conducted to rigorously assess the causative link between immune cell types and AF. A diagram illustrating the symptoms, treatments, and current pathophysiological mechanisms of AF is provided as Figure S1, Supplemental Digital Content, https://links.lww.com/MD/Q997.

### 2.2. Sources of exposure and outcome data

In this study, we utilized a comprehensive dataset that includes immune cell types for 731 different cell types. These data were sourced from the blood cell consortium, which conducted a genome-wide association study (GWAS). The blood cell consortium dataset includes UK Biobank and international cohorts, comprising 563,085 predominantly European participants. This GWAS provides genetic variation information associated with circulating white blood cell subtypes, including leukocytes, monocytes, lymphocytes, neutrophils, eosinophils, and basophils. Additionally, the dataset includes information on lymphocyte subsets such as HLA DR+ natural killer cells, CD4+ regulatory T cells, natural killer T cells, CD4+ CD8dim T cells, CD8+ T cells, and B to investigate the causal relationship between AF and immune cell types.^[27]^ We also employed a GWAS-based AF dataset. This dataset includes 1,030,836 individuals of European ancestry (60,620 AF cases, 970,216 controls). The ricdata provided by this dataset allowed us to perform a detailed analysis of the potential causal relationship between immune cell types and AF.^[28]^

### 2.3. Statistical analysis

In this study, the selection and validation process for the instrumental variables (IVs) used in MR analysis is as follows: First, we selected genetic variants associated with immune cell types from publicly available GWAS, with a significance threshold set at *P* < 1 × 10^‐5^.^[26]^ Variants were filtered using the 1000 Genomes Project linkage disequilibrium reference panel (*R*^2^ < 0.001).^[29]^ To ensure the strength of the IVs, we conducted an *F*-statistic test (*F* > 10) to assess their strength and excluded those with weak power. For the reverse MR analysis, we applied a more stringent selection process, with a significance threshold of *P* < 5 × 10^‐8^, and excluded IVs with *F* < 10.^[30]^ Ultimately, 111 valid AF-related IVs were retained. All statistical analyses were conducted in R (version 4.0.3), with MendelianRandomization and TwoSampleMR packages. We used the random-effects inverse variance weighted (IVW) method and the weighted median method to estimate the causal relationship between immune cell types and AF.^[31]^ Additionally, to assess the heterogeneity of the IVs, we applied the Cochran *Q* test (*P* < .05) and the MR-Egger intercept test (*P* < .05) to evaluate potential pleiotropy.^[32]^ Sensitivity analyses,^[33]^ including radial MR testing, leave-one-out,^[34]^ and MR-PRESSO, validated result robustness.^[35]^ Various graphical representations, such as scatter plots, funnel plots, and forest plots, were used to evaluate the influence of outliers, the robustness of the analyses, and the stability of IV effects. Through these steps, we ensured the transparency of the Mendelian randomization analysis and the reliability of causal inference.

## 3. Results

### 3.1. The causal relationship between immune cell types and AF

**Figure 1.**
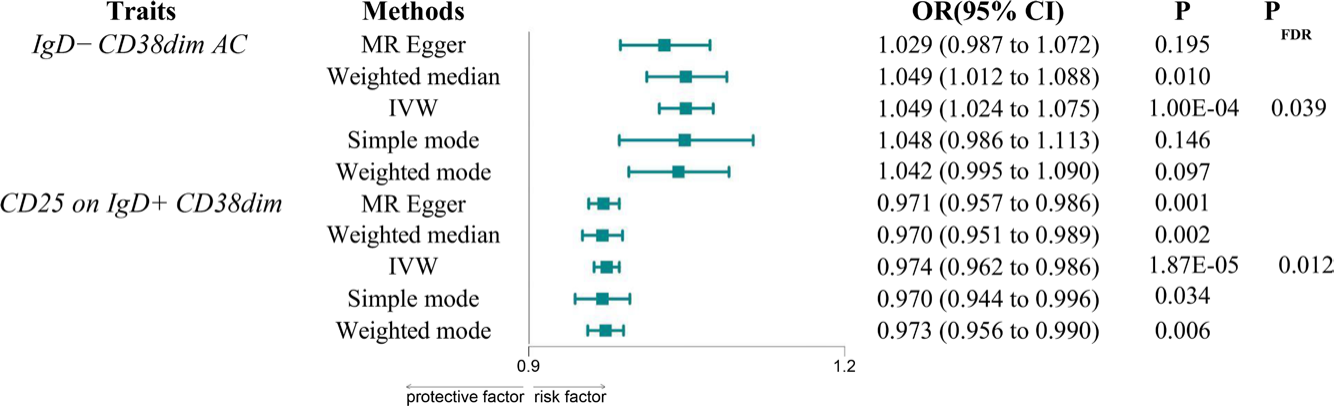
The forest map shows a causal relationship between immune cell types and AF. AF = atrial fibrillation, CI = confidence interval, IVW = inverse weighted variance.

As primary analytical approaches, we utilized 2-sample MR analysis and the IVW method to explore the causal relationship between immune phenotypes and AF. All selected single nucleotide polymorphisms had *F*-statistics > 10, confirming their robustness as instrumental variables. Post false discovery rate (FDR) adjustment (PFDR < 0.05), 2 immune phenotypes were identified as causally associated with AF. Igd-cd38dim AC was found to be a risk factor for AF (odds ratio = 1.049, 95% confidence interval =1.024–1.075, *P* = .0001, PFDR = 0.039), while CD25 on IgD^+^CD38dim was identified as a protective factor for AF (odds ratio = 0.974, 95% confidence interval = 0.962–0.986, *P* = 1.87E‐05, PFDR = 0.012) (Fig. 1). Cochran *Q* test indicated no significant heterogeneity, and the MR-Egger intercept suggested no pleiotropy (*P* > .05) (Supplementary Tables S1 and S2, Supplemental Digital Content, https://links.lww.com/MD/Q998). The robustness of our findings was further validated through forest plots, funnel plots, scatter plots, and sensitivity analysis plots (Figures S2 and S3, Supplemental Digital Content, https://links.lww.com/MD/Q997).

### 3.2. Causal relationship between AF and immune cell types

To ascertain how the progression of AF influences the human immune mechanisms, we conducted a MR analysis to investigate the causal effects of AF on immune cell levels. No significant causal associations remained after FDR correction (PFDR > 0.05).

## 4. Discussion

This study utilized 2-sample MR analysis to investigate the causal relationship between immune cell types and AF, highlighting the potential role of immune-related phenotypes in the pathogenesis of AF. Traditional Chinese medicine (TCM) has long emphasized the holistic regulation of the human body and the balance of Yin and Yang, which resonates with modern concepts of immune homeostasis and inflammatory modulation. From a systems perspective, AF is not only an electrophysiological disorder but also a manifestation of systemic imbalance involving inflammation, oxidative stress, and neurohumoral activation. TCM therapies, through herbal formulas, acupuncture, and integrated approaches, have been reported to modulate immune function, attenuate oxidative stress, and improve microcirculation, thus offering potential adjunctive benefits for AF management. Increasing evidence suggests that TCM exerts immunoregulatory effects across a spectrum of human diseases, providing a strong theoretical foundation for its application in cardiovascular disorders. For instance, TCM-derived compounds have been demonstrated to be useful for various human diseases, such as brain diseases,^[9]^ infectious diseases,^[36–40]^ bone disorders,^[41–43]^ cancer,^[44–47]^ and even organ transplantation.^[48]^ These studies collectively underscore the multi-target and multi-pathway nature of TCM interventions. Integrating these insights with modern immunogenetic findings, such as those revealed in the present MR analysis, may help bridge traditional therapies with molecular immunology to design novel AF treatment paradigms emphasizing immune balance and systemic regulation.

In the present study, the analysis identified significant causal associations between 2 immune phenotypes and AF, providing critical insights into the mechanisms by which immune cells contribute to AF and their potential as therapeutic targets. Additionally, reverse MR analysis was performed to assess the impact of AF on immune cell levels, revealing no significant causal relationships after multiple testing correction. These findings provide new insights into AF pathophysiology. Increasing evidence suggests significant alterations in the immune system during AF, interacting with the cellular and environmental elements involved in the initiation and perpetuation of AF. Immune remodeling is a pervasive process throughout the onset and maintenance of AF.^[49,50]^ This process not only induces changes in myocardial electrophysiology, structure, and neurology but also leads to AF-related pathological alterations, including fibrosis, thereby playing a crucial role in the pathophysiology of AF.^[51,52]^ Observational studies indicate the presence of immune cell-mediated atrial remodeling and inflammation in atria affected by AF, a phenomenon not observed in atria without AF.^[7]^ MR analysis is commonly employed to elucidate potential causal relationships between risk factors and diseases.This investigation employs an integration of extensive individual-level and aggregated GWAS datasets to elucidate the role of immune cells in the etiology and progression of AF from a genetic standpoint. The study offers compelling evidence that immune cells contribute to the risk of AF, utilizing a comprehensive genetic framework based on large-scale GWAS aggregated data. Using single nucleotide polymorphisms as instruments, 2-sample MR identified 2 immune cell types significantly associated with AF, namely IgD^‐^CD38dim B cells and CD25 on IgD^+^CD38dim B cells, with the risk of AF. This is the first MR analysis to explore causal associations between multiple immune phenotypes and AF.

Elevated IgD^‐^CD38dim AC levels are associated with increased AF risk. This supports the hypothesis that certain immune cell subtypes contribute to AF by promoting inflammation and structural remodeling of atrial tissue. The elevated activity of IgD^‐^CD38dim AC may exacerbate atrial fibrosis and electrical remodeling by releasing pro-inflammatory cytokines, disrupting normal conduction pathways, and creating an arrhythmogenic substrate. These findings align with studies emphasizing inflammation’s role in AF, where cytokines such as interleukin-6 and tumor necrosis factor-alpha drive atrial remodeling and arrhythmogenesis.^[53]^ Additionally, IgD^‐^CD38dim AC might activate other immune cell subsets, such as macrophages and T cells, amplifying local inflammatory cascades and worsening AF progression. In contrast, CD25 expression on IgD^+^CD38dim cells exhibited a protective effect against AF, potentially linked to its role in regulatory T cells (Tregs).^[54]^ Tregs play a crucial role in maintaining immune homeostasis by suppressing excessive inflammation through anti-inflammatory cytokines like interleukin-10. Impaired Treg function or reduced Treg numbers have been associated with an increased risk of AF.^[53]^ Therefore, enhancing Treg activity or restoring their population may represent a promising immunomodulatory approach to mitigate AF risk. The robust statistical framework employed in this study, comprising *F*-statistic tests, heterogeneity assessments, and sensitivity analyses, ensures the validity of our conclusions. By using stringent criteria for instrument selection and leveraging advanced MR methods, such as IVW and weighted median analyses, we minimized potential biases arising from confounding factors or pleiotropy. Sensitivity analyses, including leave-one-out and MR-PRESSO tests, further reinforced the robustness of our findings, while graphical tools provided a comprehensive evaluation of instrumental variable stability.

Our reverse MR analysis aimed to evaluate whether AF exerts a causal influence on immune cell levels. However, after correction for multiple testing, no significant causal relationships were identified. This finding suggests that although AF involves complex pathological processes—such as oxidative stress, atrial stretch, and tissue remodeling—these mechanisms may primarily act at the local cardiac level without inducing systemic alterations in immune cell composition. Based on the causal associations identified between specific immune phenotypes and AF in the present study, several practical recommendations can be proposed for clinical application. For individuals at high risk of developing AF, the frequency of IgD^‐^CD38^dim B cells and the expression level of CD25 on IgD^+^CD38^dim B cells could be integrated into risk stratification frameworks. If the frequency of IgD^‐^CD38^dim B cells is elevated or CD25 expression on IgD^+^CD38^dim B cells is reduced, clinicians should consider intensifying ambulatory electrocardiogram monitoring. In addition, priority should be given to lifestyle interventions, such as weight management and smoking cessation, to reduce inflammatory triggers and mitigate AF risk associated with dysregulated immune phenotypes. For patients with established AF who exhibit suboptimal responses to standard pharmacotherapy, additional assessment of immune phenotypes may be warranted. If a marked elevation in IgD^‐^CD38^dim B cell frequency is detected, clinicians might cautiously consider short-term adjunctive use of anti-inflammatory agents after evaluating potential benefits and risks. In cases where CD25 expression on IgD^+^CD38^dim B cells is insufficient, assessment of Treg function may provide further insight. This could inform the exploration of immunomodulatory strategies aimed at enhancing Treg activity, thereby restoring immune homeostasis and potentially reducing AF recurrence.

### 4.1. Limitations and future directions

This study has several noteworthy limitations. First, the dataset used primarily consists of individuals of European ancestry, which may constrain the generalizability of our findings to other populations. To enhance the broader applicability of these results, future research should include more diverse cohorts to mitigate potential population structure biases that may influence the extrapolation of our conclusions. Second, our analysis relies on GWAS data, which inherently carries the risk of exposure and outcome measurement biases. Moreover, there are inherent limitations in using publicly available datasets, including potential differences in data quality, population structure, and phenotypic definitions.^[55,56]^ Despite conducting extensive sensitivity analyses to address potential pleiotropy, residual confounding effects cannot be entirely ruled out. Furthermore, while this study provides evidence supporting a causal relationship, the lack of experimental validation limits the mechanistic interpretation of our findings. Future investigations integrating RNA sequencing or single-cell analysis could offer more direct insights into the underlying biological pathways. To ensure the robustness of our findings, independent validation using additional datasets is necessary. The molecular mechanisms by which immune phenotypes influence AF remain insufficiently understood and warrant further exploration. Experimental models incorporating multi-omics approaches may provide more definitive causal evidence and help identify novel molecular targets for therapeutic intervention. Moreover, large-scale clinical trials evaluating the efficacy of immune-targeted therapies in AF patients are essential to facilitate the translation of these findings into clinical practice, ultimately improving patient outcomes.

## 5. Conclusion

This study provides strong evidence linking immune phenotypes to AF pathogenesis. IgD^‐^CD38dim AC increased AF risk, whereas CD25 expression on IgD^+^CD38dim cells had protective effects. Our results emphasize the potential of immune modulation as a therapeutic avenue for AF, warranting further validation in experimental and clinical settings. Future research integrating genetic, molecular, and clinical data will be critical in advancing the precision medicine approach to AF management.

## Acknowledgments

I sincerely appreciate the shared public database resources, which have been invaluable to my research and enhanced the quality of this study.

## Author contributions

**Funding acquisition:** Jian Shi.

**Investigation:** Luofei Huang, Jian Shi.

**Software:** Quanzhi Lin, Jian Shi.

**Supervision:** Han Li.

**Writing – original draft:** Luofei Huang.

**Writing – review & editing:** Han Li, Quanzhi Lin, Jian Shi.

## Supplementary Material




