# Supplementary material for: Causal role of 731 immune cell types in atrial fibrillation: A bidirectional two-sample Mendelian randomization study
Source: Medicine (Baltimore). 2025 Dec 19;104(51):e46767. doi: 10.1097/MD.0000000000046767 (PMC12727287; doi:10.1097/MD.0000000000046767)
Supplement: Supplementary file 1 [file medi-104-e46767-s001.pdf]

Atrial Fibrillation (AF)

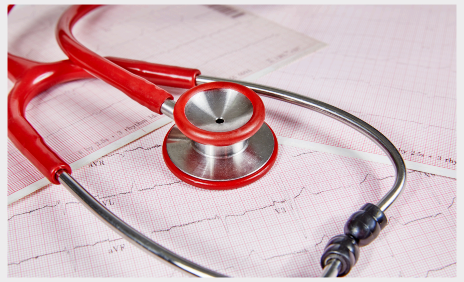

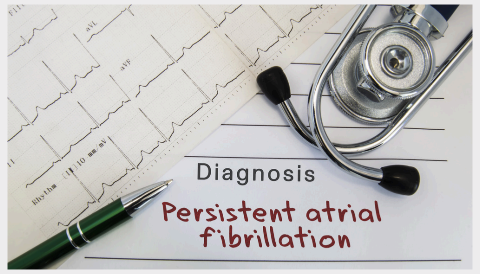

Palpitations
(rapid/irregular beat)

Dyspnea
(rest/activity)

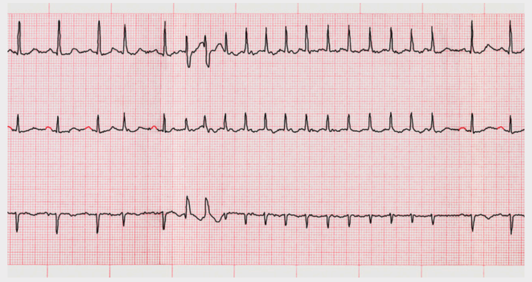

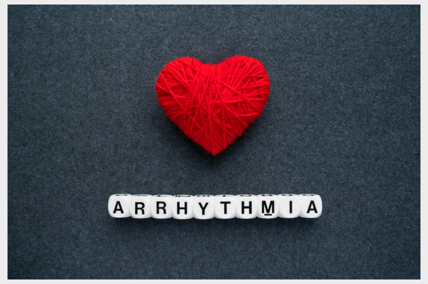

Fatigue
/weakness

Chest discomfort

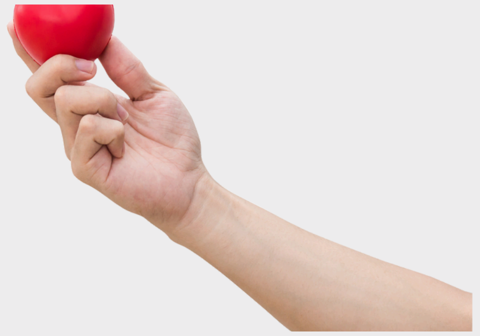

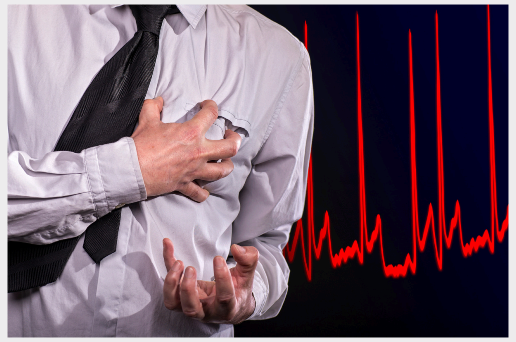

Asymptomatic AF

Syncope/presyncope

Evidence-Based Treatment and Prevention

Thromboembolism Prevention

Anticoagulants (DOACs/VKAs);
LAAC (high bleeding risk)

Heart Rate Control

Meds (β-blockers, CCBs, digoxin);
AV ablation + pacemaker

Rhythm Control

Cardioversion; antiarrhythmics (Ic/III);
catheter ablation

Pathophysiology

Electrical Remodeling

Shortened atrial refractory period,
abnormal ion channels

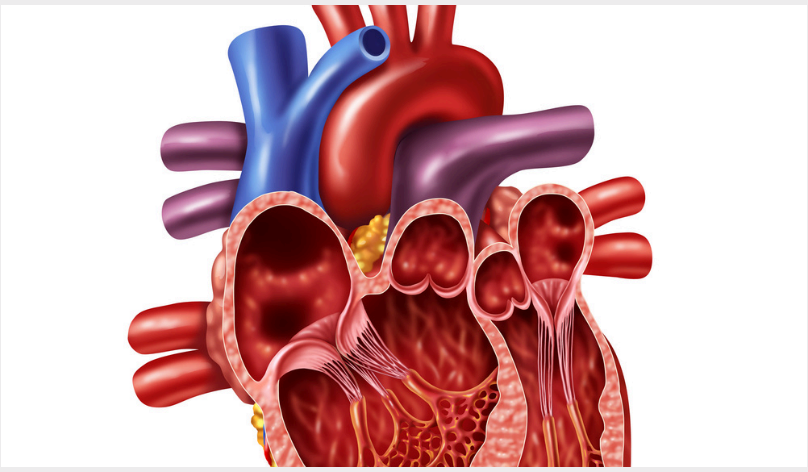

Structural Remodeling

Atrial fibrosis/hypertrophy (HTN, HF,
aging-driven)

Inflammatory Remodeling

Immune infiltration,
proinflammatory cytokines
(mediates remodeling; study focus)

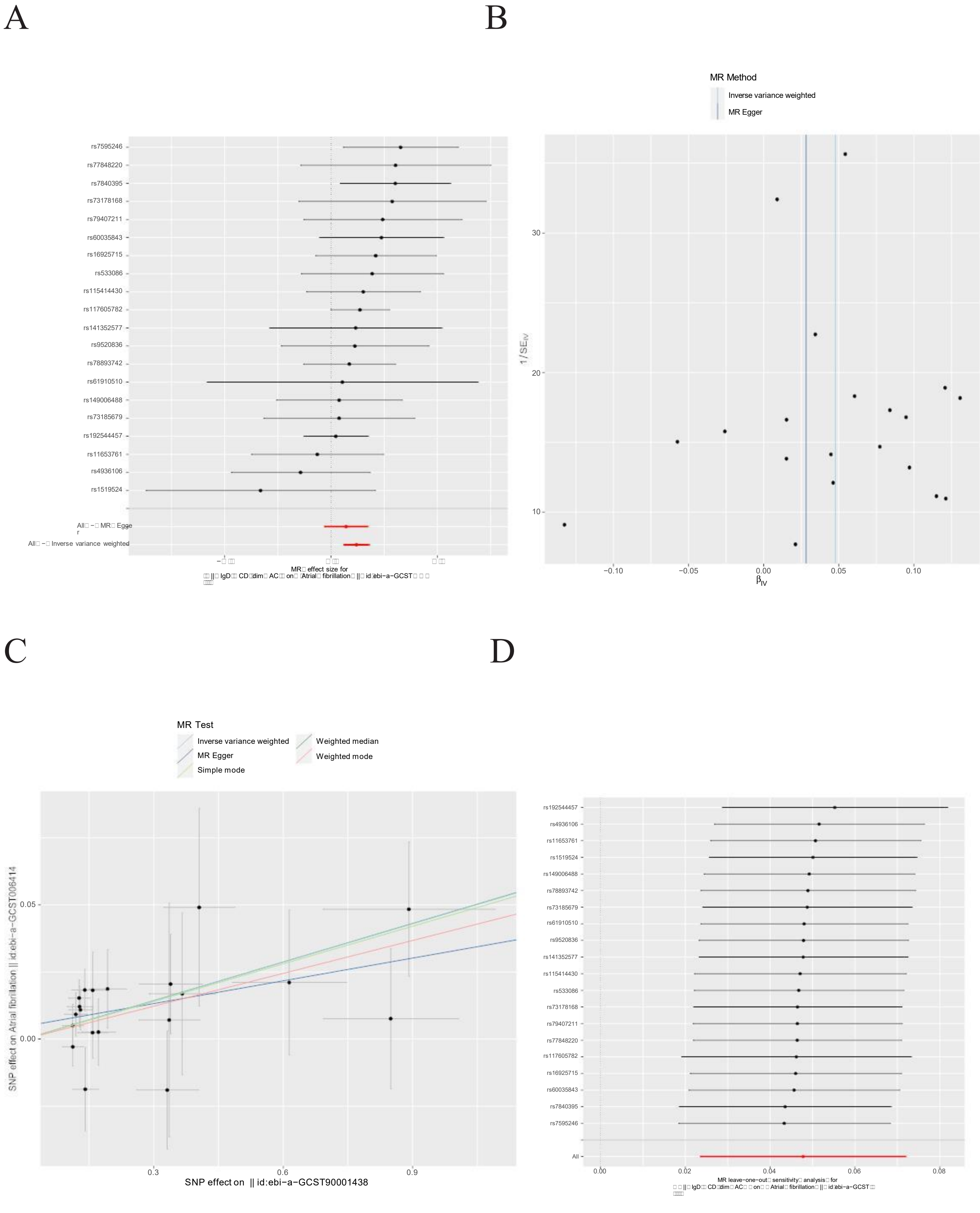
A
B
C
D
rs7595246
rs77848220
rs7840395
rs73178168
rs79407211
rs60035843
rs16925715
rs533086
rs115414430
rs117605782
rs141352577
rs9520836
rs78893742
rs61910510
rs149006488
rs73185679
rs192544457
rs11653761
rs4936106
rs1519524
All - MR Egger
All - Inverse variance weighted
MR effect size for
IgD CD dim AC on Atrial fibrillation || id:ebi-a-GCST
MR Method
Inverse variance weighted
MR Egger
1/SE_IV
β_IV
30
20
10
−0.10
−0.05
0.00
0.05
0.10
MR Test
Inverse variance weighted
MR Egger
Simple mode
Weighted median
Weighted mode
SNP effect on Atrial fibrillation || id:ebi-a-GCST006414
SNP effect on || id:ebi-a-GCST90001438
0.05
0.00
0.3
0.6
0.9
rs192544457
rs4936106
rs11653761
rs1519524
rs149006488
rs78893742
rs73185679
rs61910510
rs9520836
rs141352577
rs115414430
rs533086
rs73178168
rs79407211
rs77848220
rs117605782
rs16925715
rs60035843
rs7840395
rs7595246
All
0.00
0.02
0.04
0.06
0.08
MR leave-one-out sensitivity analysis for
IgD CD dim AC on Atrial fibrillation || id:ebi-a-GCST

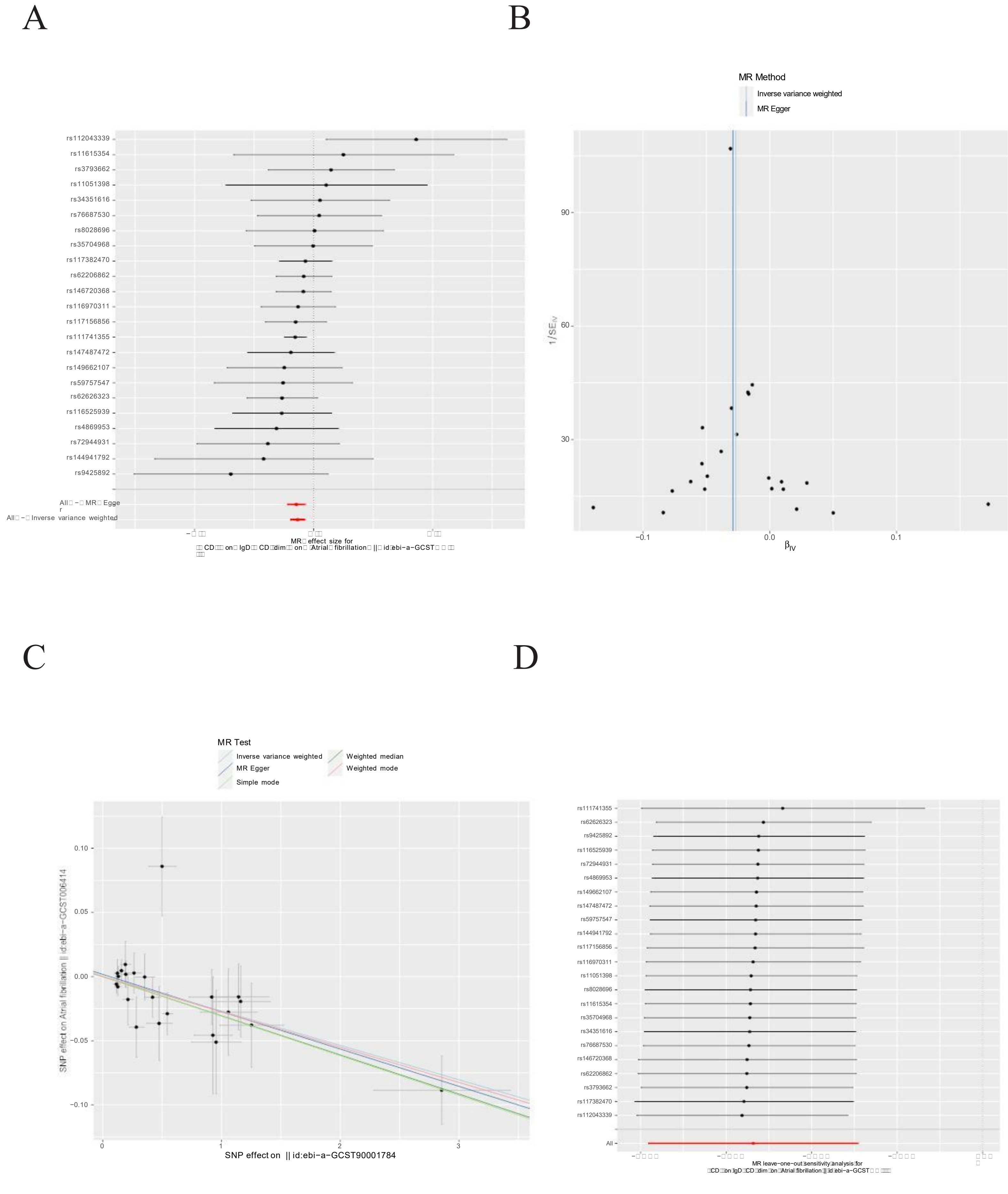
A
B
C
D
MR Method
Inverse variance weighted
MR Egger
1/SE
β
MR Test
Inverse variance weighted
MR Egger
Simple mode
Weighted median
Weighted mode
SNP effect on Atrial fibrillation || id:ebi-a-GCST006414
SNP effect on || id:ebi-a-GCST90001784
All - Inverse variance weighted
All - MR Egger
MR effect size for
MR leave-one-out sensitivity analysis for
rs112043339
rs11615354
rs3793662
rs11051398
rs34351616
rs76687530
rs8028696
rs35704968
rs117382470
rs62206862
rs146720368
rs116970311
rs117156856
rs111741355
rs147487472
rs149662107
rs59757547
rs62626323
rs116525939
rs4869953
rs72944931
rs144941792
rs9425892
